# Identifying the Gaps Between Public Health Training and Practice: A Workforce Competencies Comparative Analysis

**DOI:** 10.3389/ijph.2022.1605303

**Published:** 2022-12-22

**Authors:** Osnat Bashkin, Robert Otok, Ori Kapra, Kasia Czabanowska, Paul Barach, Orna Baron-Epel, Keren Dopelt, Mariusz Duplaga, Lore Leighton, Hagai Levine, Fiona MacLeod, Yehuda Neumark, Stephanie Paillard-Borg, Theodore Tulchinsky, Zohar Mor

**Affiliations:** ^1^ Department of Public Health, Ashkelon Academic College, Ashkelon, Israel; ^2^ Association of Schools of Public Health in the European Region (ASPHER), Brussels, Belgium; ^3^ School of Health Professions Education, Faculty of Health, Medicine and Life Sciences, Maastricht University, Maastricht, Netherlands; ^4^ Jefferson College of Population Health, Philadelphia, PA, United States; ^5^ Interdisciplinary Research Institute for Health Law and Science, Sigmund Freud University Vienna, Vienna, Austria; ^6^ School of Public Health, Faculty of Social Welfare and Health Sciences, University of Haifa, Haifa, Israel; ^7^ Department of Health Policy and Management, School of Public Health, Faculty of Health Sciences, Ben Gurion University of the Negev, Beer-Sheva, Israel; ^8^ Department of Health Promotion and e-Health, Institute of Public Health, Faculty of Health Sciences, Jagiellonian University Medical College, Krakow, Poland; ^9^ The Israeli Association of Public Health Physicians (IPAPH), Israeli Medical Association, Ramat-Gan, Israel; ^10^ School of Public Health, University College Cork, Cork, Ireland; ^11^ Hadassah Braun School of Public Health and Community Medicine, Faculty of Medicine, Hebrew University of Jerusalem, Jerusalem, Israel; ^12^ Department of Health Sciences, The Swedish Red Cross University (SRCU), Huddinge, Sweden

**Keywords:** competencies, healthcare management, public health workforce, public health training, essential public health functions

## Abstract

**Objectives:** The study aimed to generate insights on how best to enhance the compatibility between Public Health training program competencies and the implementation of competencies required by employers to address current and emerging public health needs.

**Methods:** A survey adapted from the WHO-ASPHER Competency Framework for the Public Health Workforce was conducted online among Israeli public health managers from August to November 2021. The survey was formulated to mirror Essential Public Health Operations. Forty-nine managers participated (37.6% response rate) in an assessment of 44 public health competencies and the core organizational public health operations.

**Results:** Analysis of Essential Public Health Operations revealed a notably high deficiency reported for Advocacy Communication and Social Mobilization for health competencies. Collaborations and Partnership and, Leadership and System Thinking were the most reported insufficient competencies, particularly in health departments and research institutes. Governmental offices reported Organizational Literacy and Adaptability competencies being deficient. Deficiencies were more impactful as the level of expertise increased.

**Conclusion:** There is a clear need for public health professionals to acquire versatile and innovative competencies in response to the ever-changing health threats.

## Introduction

The coronavirus disease 2019 (COVID-19) pandemic continues to threaten and strain healthcare systems and their supply chains, with high volumes of critically ill patients and an ongoing shortage of healthcare providers. The central roles played by Public Health (PH) systems and services in responding to, and supporting the management of the COVID-19 pandemic have highlighted the need to maintain a highly competent PH workforce (PHW). PH workforces across the world have had to react and adjust swiftly to the pandemic in addition to performing ongoing PH responsibilities (e.g., monitoring of infectious and chronic diseases, routine childhood vaccine programs) and controlling other emerging health threats [1]. Yet, not enough is known about PH workforce competencies assessment.

Prior studies have highlighted several organizational frameworks that serve as reference tools for PH academic training and ongoing professional development programs [2]. The Council on Education for Public Health [CEPH] specifies PH competencies such as evidence-based approaches to public health, planning and management to promote health, leadership, communication, interprofessional practice, systems thinking, and more [3]. Competency frameworks help identify gaps in knowledge, skills, and attitudes between training and practice requirements and can shape future competency-based educational programs for PH professionals [4].

The World Health Organization (WHO) Regional Office for Europe and the Association of Schools of Public Health in the European Region (ASPHER), defines ten competency categories that focus on PH content and contexts, relations and interactions, performance, and achievement [5]. The WHO-ASPHER Competency Framework for the Public Health Workforce in the European Region (CFPHW) provides standardization and consistent definitions for the competencies required by PH professionals, such as observing and analyzing health patterns and identifying at-risk and target groups for interventions, analyzing the cost-effectiveness of interventions, and providing a critical interpretation of research documentation.

The WHO Regional Office for Europe published an updated version of the ten “Essential Public Health Operations” (EPHOs) [6]. The EPHOs are used to assess public health capacities and services and define the needed competency profile of PH graduates [7]. Studies have shown, that despite extensive efforts to define PH professional competencies in academic programs, PH graduates do not possess the real-world needed competencies as measured by the EPHOs [8, 9]. We hypothesized about the differences in the assessed competencies of the PH workforce by different healthcare employers. This study contributes to the ongoing discussion about core competency measures as it relates to the public health workforce.

## Methods

### Participants

A cross-sectional survey of Israel’s entry, mid-, and senior-level PH managers was conducted. An established team (consisting of in-country experts in consultation with European Union partners) of experienced researchers in survey design, public health and health management, clinicians, PH, and health promotion practitioners designed, piloted, and analyzed the survey results.

The study was carried out through a multinational Erasmus + Capacity Building in Higher Education funded project—“Sharing European Educational Experience in Public Health for Israel (SEEEPHI): harmonisation, employability, leadership, and outreach” [10, 11]. We aimed to take advantage of established tools aimed at building PHW capacity in the European Region, adapting them to the Israeli context, to generate information that can enhance the compatibility between the qualifications of PH training program graduates in Israel and the practical competencies needed to address Israel’s emerging PH needs. Part of the results presented is published in a full report of work package 2 of the SEEEPHI project on ASPHER website [11].

### Overview of the Israeli Healthcare System

Israel is a relatively young society of 74 years with a diverse population of different ethnic, religious, cultural, and socioeconomic backgrounds, requiring cultural and structural sensitivity when addressing public health needs [10]. Yet, workforce competencies have not been assessed for the Israeli public healthcare system. The structure of health services in Israel combines mandatory state insurance with additional supplementary non-profit healthcare plans. Every citizen or permanent resident of Israel can choose from four competing nationwide Health Maintenance Organizations (HMOs). The HMOs must provide their members access to a statutory benefits package called a “Health Basket” [12]. The Israel Ministry of Health (MoH) regulates the healthcare system and owns and operates about half of acute care hospital beds, although these hospitals increasingly operate autonomously. The largest HMO operates another third of the hospital beds, and the remainder is operated through a mix of non-profit and for-profit organisations [12]. The Israeli Association of Public Health Physicians is responsible for setting the competencies, training needs and programs, and qualifications needed in Israel.

### Study Measures

The questionnaire was adapted from the WHO-ASPHER Competency Framework for the Public Health Workforce in the European Region, considering the specificity of the Israeli public health system [5]. The questionnaire was pretested by three of the researchers deeply familiar with the Israeli PH system using a modified Delphi method process to establish consensus. The modified Delphi method is an effective process for determining expert group consensus when there is little evidence and expert opinion is important [13]. The modified Delphi method consisted of three rounds of zoom meetings. The original English version framework, consisted of 84 competencies, was examined in the first round. During the meeting, each participant was asked to respond “yes/no” on the relevance for inclusion of each competency and suggested edits to the competencies. Competencies with no consensus were deleted or edited to achieve the observations of the participants. In addition, the scale of answers was also discussed to examine its suitability for the study. It was decided to develop a new ranking of answers to achieve accuracy in data collection.

In the second round, the modified English version of the questionnaire consisting of 60 competencies was discussed and sought a “yes/no” response regarding the edits made in the previous round. A consensus regarding the final version of the survey instrument was reached based on the responses obtained during round two, and the questionnaire was translated into Hebrew.

In the third round, a Hebrew version questionnaire consisting of 44 competencies was discussed regarding approval of the survey. At the end of the procedure, the questionnaire was reviewed and finalized.

The questionnaire included two parts (Supplementary Material SA):

1. The first part included ten demographic questions regarding the respondent’s age, gender, profession, employment variables, organization, subordinates, and levels of education. In addition, respondents were asked to indicate which of the ten EPHOs were most pertinent to their work and the organization they work in.
2. The second part comprised 44 PH competencies divided into ten categories mapped to the ten EPHOs. For each competency, respondents were asked to rate on a three-point scale: 1) if they have enough workers to fulfill the competency, 2) if more personnel with the described competency are needed, and 3) if the competency is or is not relevant to their organization. A three-point scale was presented corresponding to the three levels of workforce expertise–competent, proficient, and expert. The reliability of items in the ten categories ranged from *α* = 0.56 to *α* = 0.86.

### Procedure

The survey was conducted with full-time Israeli PH and healthcare systems employees whose position would require the use of public health core competencies. The approach followed a purposeful sampling strategy to identify and select an information-rich sample of participants. An online questionnaire was distributed in August 2021 to 130 high-and mid-level PH managers representing various healthcare organizations: regional health departments, headquarters of governmental offices, health research institutes, hospitals, HMOs, and non-governmental organizations (NGOs). Two electronic mail (e-mail) reminders were sent 1 and 2 months after the initial contact, respectively. The survey was closed in November 2021.

### Statistical Analysis

We measured reliability using Cronbach’s alpha. Participants were divided into five groups to analyze the survey data: 1) managers who work in regional health departments (25, 51%), 2) managers who work in the headquarters of governmental offices (14, 29%), 3) managers who work in research institutes including universities (6, 12%), 4) managers who work in hospitals and HMOs (14, 28%), and 5) managers who work in NGOs (7, 14%). We calculated percentages of responses to test differences among organizations for each of the ten categories of PH competencies and each of the ten EPHOs. Each of the WHO-ASPHER framework competencies is linked to one or more of the EPHOs. Therefore, a deficiency rate for each EPHO was calculated by subtracting the percentages of respondents who noted they have enough workers to fulfill the competency from the percentages of respondents who reported more personnel were needed with the described competency. Data analysis was carried out using IBM SPSS Statistics 25.0 software.

### Ethics

The study was approved by the Ashkelon Academic College Ethics Committee (Approval # 31-2021). Participants gave informed consent for inclusion in the study and were informed about the procedures planned for anonymity, data protection, and privacy.

## Results

### Demographics

The study consisted of 49 managers who responded to the questionnaire (37.6% response rate). Among the participants, 45% were medical doctors, 51% had a master’s degree in PH, and 20% had a Ph.D. degree (Table 1). Most participants were employed at regional health departments or governmental offices (51%), followed by MoH headquarters and other governmental offices (29%). Respondents were predominantly female. Reported experience in the main workplace was the highest among HMOs (19.2 years) and NGOs (19.9 years).

**TABLE 1:** Characteristics of the sample of public health managers who responded to the survey (N = 49) (Identifying the Gaps Between Public Health Training and Practice: A Workforce Competencies Comparative Analysis, Ashkelon, Israel, 2022).

Characteristic			
	N (%)	Women, %	Reported experience in main workplace, mean years (SD)
Total Respondents	49	67.3	17.1 (9.5)
Educational Level			
Bachelor’s Degree	9 (18.4)	55.6	21.7 (5.9)
Master’s Degree	21 (51.0)	76.2	15.8 (11.0)
Doctor of Philosophy (PhD)	10 (20.4)	60.0	15.6 (8.2)
Doctor of Medicine (M.D.)	22 (44.9)	59.1	16.6 (9.2)
Organizations			
Hospital	8 (16.3)	75.0	15.1 (8.2)
HMO	6 (12.2)	33.3	19.2 (3.4)
Regional Health Department	25 (51)	60.0	18.2 (9.1)
Governmental offices, including the Ministry of Health Headquarters	14 (29)	64.3	15.3 (7.9)
Health research institute	6 (12.2)	83.3	13.8 (9.9)
NGO: Non-Profit Organization, Private Organization, or Other	7 (14.2)	71.4	19.9 (11.1)

HMO, health maintenance organization; NGO, Non-Governmental Organization.

### Essential Public Health Operations

The analyses of the survey results revealed that all EPHOs were relevant to PH and healthcare organizations (Table 2). However, some distinctions were identified between the levels of relevance to the different organizations. For example, participants affiliated with research institutes reported that EPHOs 1—*Surveillance of population health and wellbeing*, and 10—*Advancing public health research to inform policy and practice*, were more relevant to their core function than respondents from other organizations. PH professionals that worked in health departments and NGOs indicated that EPHO 3—*Health protection, including environmental occupational, food safety, and others*, was relatively more essential to fulfill the professional responsibilities of their organization. Those who worked in hospitals, HMOs and NGOs indicated that EPHO 5—*Disease prevention, including early detection of illness*, was more relevant for their core functions. EPHO 8—*Assuring sustainable organizational structures and financing*, was ranked among the least relevant EPHOs, particularly among hospitals, HMOs, and NGO workers.

**TABLE 2:** Rates of Essential Public Health Operations as core functions by organizations (%) (Identifying the Gaps Between Public Health Training and Practice: A Workforce Competencies Comparative Analysis, Ashkelon, Israel, 2022).

Organizations	Hospitals, HMOs (N = 13)	Health departments (N = 25)	Government (N = 14)	Research institutes (N = 6)	NGOs (*n* = 7)
EPHO1: Surveillance of population health and wellbeing	53.8	52	50	66.7	57.1
EPHO2: Monitoring and response to health hazards and emergencies	30.8	56	42.9	33.3	85.7
EPHO3: Health protection including environmental occupational, food safety and others	15.4	88	42.9	50	85.7
EPHO4: Health promotion including action to address social determinants and health inequity	46.2	48	35.7	50	71.4
EPHO5: Disease prevention, including early detection of illness	61.5	52	57.1	50	85.7
EPHO6: Assuring governance for health and wellbeing	23.1	32	28.6	33.3	28.6
EPHO7: Assuring a sufficient and competent health workforce	46.2	32	42.9	50	57.1
EPHO8: Assuring sustainable organizational structures and financing	15.4	20	21.4	33.3	14.3
EPHO9: Advocacy communication and social mobilization for health	30.8	24	28.6	16.7	71.4
EPHO10: Advancing public health research to inform policy and practice	46.2	24	42.9	100	71.4

HMO, health maintenance organization; NGO, Non-Governmental Organization.

#### EPHOs-Related Skill Deficiencies

**FIGURE 1:**
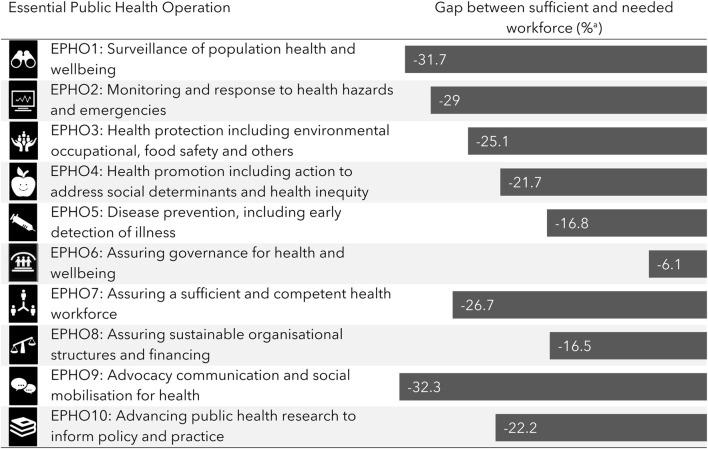
Essential Public Health Operations gaps between sufficient and needed workforce^a^ (Identifying the Gaps Between Public Health Training and Practice: A Workforce Competencies Comparative Analysis, Ashkelon, Israel, 2022).

Most of the survey’s competencies were formulated to mirror specific EPHOs, allowing an analysis of reported competencies and deficiencies as they pertain to each of the ten particular EPHOs (Figure 1). There was uniform deficiency in competencies across all EPHOs, with the reported levels between 6.1% and 31.7%. A lower deficiency of 6.1% was reported for EPHO 6—*Assuring governance for health and wellbeing*. The reported deficiency was notably high for EPHO 9—*Advocacy communication and social mobilization for health.*

### WHO-ASPHER Competency Framework

Table 3 presents the rates of the ten categories of competency deficiencies as reported by different organizational contexts, representing both the extent of deficiency and the specific category of competency shortfalls according to the WHO-ASPHER Competency Framework. The Collaborations and Partnership and, Leadership and System Thinking, were the most reported insufficient category of competencies across all organizations, particularly in health departments and research institutes. Collaboration and Partnership was the most reported insufficient category of competencies among NGOs.

**TABLE 3:** Rates of 10 categories of competency deficiencies as reported by organizations^a^ (%) (Identifying the Gaps Between Public Health Training and Practice: A Workforce Competencies Comparative Analysis, Ashkelon, Israel, 2022).

Organizations	Hospitals, HMOs (N = 13)	Health departments (N = 25)	Government (N = 14)	Research institutes (N = 6)	NGOs (*n* = 7)
Science and Practice	49.7	46.1	35.1	62.5	48.5
Promoting Health	48.4	44.5	38.6	65.6	40.6
Law, Policies and Ethics	47.5	50.7	40	66	52.1
One Health and Health Security	42.3	51.8	32.2	66	32.8
Leadership and System Thinking	46.7	62.9	40	68.1	37.7
Collaborations and Partnership	43.5	60.9	40.6	68.9	56.4
Communication, Culture and Advocacy	37.3	61.7	32.7	48.2	43.8
Governance and Resource Management	42.3	56.5	31	57.8	28.8
Professional Development and Reflective Ethical Practice	44.2	47.7	24.2	52.8	13.8
Organizational Literacy and Adaptability	48.1	56.5	45.6	58.3	23.4

HMO, health maintenance organization; NGO, Non-Governmental Organization.

^a^ Response percent averages calculated for total items per category.

The highest reported deficiencies, across nine out of the ten competency categories, were reported by research institutes, with the most pronounced shortfalls being in Science and Practice (62.5%), Promoting Health (65.6%), Law, Policy and Ethics (66%), and One Health and Health Security (66%), all included under “Content and Context” competencies, as well as competencies related to Leadership and Systems Thinking (68.1%) and Collaboration and Partnerships (68.9%).

The regional health departments also noted significant shortfalls in PH workforce skills. Deficiencies were mainly in Leadership and Systems Thinking (62.9%), Collaboration and Partnerships (60.9%), and Communication, Culture and Advocacy categories (61.7%). Within these categories, the most highly demanded competency was “Identifying and describing the environmental factors that affect public health,” followed by “Promoting projects and addressing barriers that may limit collaboration.” Compared to other organizations, the demand for Communication, Culture and Advocacy competencies was marginally higher in health departments, which mainly required the “Ability to communicate with speakers and media people, identify target audiences, and develop messages” and “Ability to be interviewed by the media” (See Supplementary Material).

Governmental offices reported the highest satisfaction overall with the competencies of their workforce, with only the Organizational Literacy and Adaptability category of competencies being more deficient (45.6%) than other categories. More specifically, the ability to deal with uncertainty and manage work-related stressful situations was the highest-demanded competency in this category. The most deficient competency was “Promoting projects and addressing barriers that may limit collaboration” (See Supplementary Material).

### Deficiencies in Levels of Public Health Expertise

Public health expertise deficiencies were identified in all categories of competencies and across the three levels of expertise, although to a lesser extent in the Professional Development and Reflective Ethical Practice themes. Generally, the deficits were more significant as the level of expertise increased. In competent-level workers, the most in-demand category of competencies was Organizational Literacy and Adaptability (indicated by 43% of the respondents), with the highest demand for the competency of “Ability to initiate and discover innovation, with unconventional solutions and thinking outside the box.” Collaboration and Partnerships was the most requested category of competencies in the proficient-level workforce (indicated by 62% of the respondents). This category was also deficient in the expert-level workforce (62%), along with Leadership and Systems Thinking competencies (63%). The most deficient competency at the proficient workforce level, mentioned by 74% of respondents, was “Promoting projects and addressing barriers that may limit collaboration.” The three most common competencies in demand by the expert workforce were: “Designing and conducting qualitative and quantitative research which builds on existing evidence, involving relevant stakeholders in the research process” (indicated by 75% of the respondents), “Leading interdisciplinary teams in public health, including external stakeholders” 72%) and “Promoting projects and addressing barriers that may limit collaboration” 72%).

## Discussion

We present a consensus-building study to identify the essential competencies considered important for the PHW as perceived by employers from various PH organizations. The study is one of the first formal studies in this area, synthesizing the cumulative learning from the literature on PH competencies coupled with front-line PH expert knowledge and needs. The study identifies gaps and specific critical competencies required by PHW to meet emerging public health challenges and disasters, such as during the COVID-19 pandemic.

The main findings of the study indicate that employers expressed a pervasive deficiency of essential competencies, a need for better-trained workers, and an urgent call for PHW to have the ability to deal with increasingly complex and diversified tasks. Advocacy communication and social mobilization for health were the least often mentioned as core PH operations of the ten EPHOs. However, this EPHO encourages modern communication methods and technologies to support leadership and public advocacy for community engagement and empowerment. In health departments and across all proficiency levels, there is a high demand for greater competencies related to Communication, Culture and Advocacy as expressed by nearly 60% of respondents. This category of competencies includes effective communication (written and verbal) with the media, scientific communication, presentation, respect for diversity and inclusiveness, historical and cultural context, advocacy, and diplomacy [5].

The discrepancy between the high demand for PH communication competencies and the low percentages of respondents mentioning EPHO 9 as a core function may reflect that *de facto*, the media profession is a distinct domain viewed as operating outside the remit of PH. However, during the COVID-19 pandemic, PH professionals, especially at the expert level, were expected to possess mature communication and advocacy competencies [14].

We found that there is a pressing need for PH professionals that are qualified to respond to current and emerging PH threats and provide appropriate responses to at-risk groups by using effective communication channels. Our findings support previous studies by Nutbeam [15] in which better PH communication is one of the professional routes to facilitate health literacy and improve the status of individuals and populations. Dopelt et al. [16] argue that good communication enhances the population’s capacity to access, understand, and use the information to reduce risk, prevent disease, promote health, and navigate and utilize health services. The findings align with a recently released WHO Information Network framework developed for Epidemics (EPI-WIN) [17]. The EPI-WIN framework emphasizes the importance of PHW competencies promoting resilience to “infodemics” among individuals and communities, dealing with mis/disinformation, and promoting self-efficacy for self-protective health behaviors.

The Organizational Literacy and Adaptability competencies were in high demand across all organizations, albeit with different foci across the three proficiency levels. This category of competencies includes technology, data management, entrepreneurship, fundraising, creativity, analysis and synthesis, digital health, social media, and the understanding of PH services and operations. In addition, competency deficiencies that appeared at all proficiency levels included a limited capacity for innovation and difficulty in “thinking outside the box.” The European Commission recently emphasized the need for advanced competencies related to learning methods and digital competencies [18].

The competencies most in demand at the proficient and expert levels, especially in research institutes and health departments, relate to knowledge regarding funding opportunities and grant application development and submission. This is indicative of a scientific culture that fosters applied research. This observation coincides with our findings that the ability to initiate and discover innovation with unconventional solutions and thinking outside the box was sought after by nearly half of the survey’s respondents. These findings highlight the ongoing need to strengthen links between PH practitioners in the field, and professionals and students from higher education institutions, confirming the results of other studies [19, 20].

Findings from this study suggest an urgent need to enhance the capacity of the Israeli PHW and should include strategies that deal with these growing competency gaps. The study highlights the needed competencies supported by the Israeli national PH strategy that supports best practices for the development of PHW and the provision of exceptional public health services in Israel.

### Limitations

Several limitations of this exploratory study must be considered in interpreting the results. First, the sample size was small, and participant bias was possible. However, we believe our results are representative due to the diverse sample of the study’s managers and the mature nature of the Israel PH system. Second, the data were collected using self-reported perceptions of competencies. Responses may be influenced based on participants’ experiences and working conditions, and the data are subject to recall and selection bias. Third, few controlled studies demonstrate the causal relationships between PH training and clinical outcomes. Since the survey is cross-sectional, the current study cannot determine causation links between PH training and PH competencies or the potential direction of the associations observed. Fourth, a Delphi method can be criticized, given the loss of subject anonymity in the voting process. We believe that subject anonymity allows for a freer expression of opinions and can reduce the effects of dominant individuals and reduce manipulation or coercion to conform to viewpoints. Consensus methods, however, provide a valuable way of identifying and measuring uncertainty in health services research [21]. Finally, our study reflects the context and distinct constraints of the Israeli healthcare delivery systems, which differ from other healthcare systems. However, the overall methodological rigor and robust research design should be applicable and generalizable to other countries.

### Conclusion and Future Directions

The COVID pandemic has reinforced the critical need for a well-qualified and proficient workforce to provide comprehensive, high-quality health services, reach minorities, reduce health inequalities, and confront emerging health challenges such as during the COVID-19 pandemic. Our analyses, using a validated survey backed by a formal expert consensus technique, allowed us to profile the work needs of a large number of PHW employers. Further work is needed to compare these findings to competencies mapping of PH training programs and develop strategies to bridge the gaps between PHW training and real-world practice. Leaders of PH services and PH higher education programs should discuss actionable ways to collaborate, redesign and better adapt educational programs to the evolving and demanding field needs. Continuous evaluation of PH competencies and those taught in PH higher education programs will prepare a competent PH workforce ready to confront new health challenges and adapt to the changing population health.

Our findings support the redesign and focus of higher education PH programs in Israel to better tailor their academic programs to meet the real-world conditions that await students upon graduation and strengthen the essential ties between academia, communities, and employers. Future work is needed to replicate these findings in other countries.
