# Supplementary material for: Identifying the Gaps Between Public Health Training and Practice: A Workforce Competencies Comparative Analysis
Source: Int J Public Health. 2022 Dec 22;67:1605303. doi: 10.3389/ijph.2022.1605303 (PMC9812945; doi:10.3389/ijph.2022.1605303)
Supplement: Supplementary file 1 [file DataSheet1.DOCX]

**Supplementary A- Public Health workforce competencies survey E-mail invitation**

Part One- Demographic questions

1. What is your highest level of education (mark more than one option if needed):1. Bachelor's degree 2? Master's degree 3. Doctorate Degree Ph.D. 4. MD 5. Other___________
2. Do you have an MPH? 1. Yes 2. No
3. What is your profession: 1. Medicine with a specialty in Public Health; 2. Medicine with another specialty; 3. Nursing; 4. Nutrition; 5. Economics/ political science; 6. Human resources; 7. Epidemiology; 8. Health Promotion; 9. Research; 10. Other_______
4. Years of employment in your workplace: ___________
5. What is the number of your direct subordinates?
6. How many of them are working in public health? 1. 25% max; 2. 25- 50%; 3. 50-75%; 4. 75% or more
7. What percentage of your workers have a degree in public health? 1. 0-25%; 2. 25-50%; 3. 50-75%; 4. 75% and up
8. Do you manage a budget as part of your on-job responsibility? 1. Yes; 2. No
9. The organisation you work at: 1. Hospital__________; 2. Clinic _____; 3. Government Office; 4. Non-profit organisation; 5. Private Company; 6. Municipality; 7. Department of Health________; 8. Public Health Services; 9. Ministry of Health headquarters; 10. University_____; 11. Research institute ________
10. The nature of your work: 1. Health management; 2. Research; 3. Clinical practice; 4. Other
11. Please select from the following list the main functions your organisation deals with. You can mark several functions or all of them:

EPHO1: Surveillance of population health and wellbeing

EPHO2: Monitoring and response to health hazards and emergencies

EPHO3: Health protection including environmental occupational, food safety and others

EPHO4: Health promotion including action to address social determinants and health inequity

EPHO5: Disease prevention, including early detection of illness

EPHO6: Assuring governance for health and wellbeing

EPHO7: Assuring a sufficient and competent health workforce

EPHO8: Assuring sustainable organisational structures and financing

EPHO9: Advocacy communication and social mobilisation for health

EPHO10: Advancing public health research to inform policy and practice

Part Two- Public Health competencies

Several competencies relevant to public health are listed below. For each competency, we will ask you to rate if you think that more workforce holding the described competency is needed, or if there is currently enough workforce holding the described competency in the organisation or unit to which you belong, in each of the three levels of expertise - Competent, Proficient, and Expert. If this competency is not relevant at all in your organisation, please check "This competency is not relevant in the unit."

1. **Science *&* Practice**
   1. Recognition of the features of the demographic structure the the society/community in Israel and understanding the process of demographic change and its implications for public health
   2. Description of the factors influencing morbidity and mortality in the population in the area of deployment of the unit
   3. Effective use of vital statistics and health indicators to assess the state of health of the population, including at-risk groups
   4. Evaluation of community-based health needs
   5. Designing and conducting qualitative and quantitative research builds on existing evidence, involving relevant stakeholders in the research process
   6. Understand the health system structure, governance, funding mechanisms, and how healthcare services are organised
   7. Knowledge of guidelines regarding disaster control and prevention of pandemics
   8. Participation in drafting guidelines regarding disaster control and prevention of pandemics
2. **Promoting health**
   1. Development of health promotion programs designed to improve the community's health and quality of life
   2. Presentation and dissemination of information related to the promotion of health in the community (such as encouraging proper nutrition, physical activity, cessation of smoking, reduction in alcohol consumption)
   3. Recognition and consideration of the leading factors for inequality in health
   4. Knowledge of various risk behaviors and addressing their consequences (such as drug use, unprotected sex, smoking, and alcohol consumption)
3. **Law, Policy & Ethics**
   1. Recognition, understanding, and application of health protocols, laws, and procedures
   2. Understand principles and concepts in public health and preparing background documents for discussion of administrative, legal, and social issues related to public health
   3. Actively participating in implementing policies that ensure the provision of equitable health services
4. **One Health & Health Security**
   1. Understand the significance of the One Health approach and its impact on the health status of the population
   2. Knowledge of International Health Regulations (IHR)
   3. Development of partnerships at the international level
   4. Understand and promote the safety, health, and wellbeing of employees in the workplace
   5. Knowledge of the practical principles of food safety
   6. Knowledge of the practical principles of healthy eating
   7. Knowledge of vaccine-preventable diseases and vaccination encouragement techniques
   8. Identify and describe environmental factors that affect public health
5. **Leadership & Systems Thinking**
   1. Encourage and motivate others to promote a shared vision and outline organisational goals
   2. Ability to serve as a model to emulate, build trust, and demonstrate sensitivity to the belief system and values ​​of the other
   3. Lead interdisciplinary teams in public health, including external stakeholders
   4. Promote change (behavioral and cultural) in the organisation, in communities and in individuals
6. **Collaboration & Partnerships**
   1. Maintain working relationships with stakeholders in interdisciplinary and cross-sectoral projects to improve health services and achieve public health goals
   2. Maintain working relationships with other departments in the Ministry of Health /hospitals/HMOs and with other agencies, such as local authorities, the Standards Institute, the Central Bureau of Statistics, other government ministries, the third sector, and relevant parliamentary committees.
   3. Promote projects and address barriers that may limit collaboration
7. **Communication, Culture & Advocacy**
   1. Ability to communicate with speakers and media, identify target audiences, and develop messages
   2. Promote health messages through media and social marketing to different audiences and adapt the messages so that they are culturally appropriate
   3. Ability to be interviewed in the media
8. **Governance & Resource Management**
   1. Manage employees efficiently, by providing clear instructions regarding task responsibilities, ensuring training, adequate resources, and providing regular performance feedback
   2. Effective planning of the assignment of work tasks in order to achieve the goals set by the organisation
   3. Detail job descriptions to promote staff absorption, conduct interviews and evaluate candidates
   4. Knowledge of the principles of economic thinking in public health
   5. Ability to apply economic principles in public health
9. **Professional Development *&* Reflective Ethical Practice**
   1. Knowledge of the ethical codes relevant to their work
   2. Ability to initiate activities for the professional development and advancement of the employees in the organisation
10. **Organisational Literacy & Adaptability**
    1. Ability to initiate and discover innovation with unconventional solutions and think outside the box
    2. Ability to deal with uncertainty and manage work-related stressful situations
    3. Ability to manage time well - allocating time frames for the fulfillment of tasks and working with deadlines
    4. Awareness and knowledge in applying available funding\ sources, development, and submission of applications and grants for projects and calls for projects
